# Single Anastomosis Sleeve Jejunal Bypass in Acute Mesenteric Artery Syndrome: A Case Report

**DOI:** 10.7759/cureus.60971

**Published:** 2024-05-24

**Authors:** Arno Talboom, Els Van Dessel

**Affiliations:** 1 General Surgery, University of Antwerp, Antwerp, BEL; 2 Abdominal Surgery, Gasthuiszusters Antwerpen, Antwerp, BEL

**Keywords:** sasj, sleeve gastrectomy, single anastomosis, gastric necrosis, superior mesenteric artery syndrome, wilkie syndrome, acute abdominal surgery

## Abstract

Superior mesenteric artery syndrome is a rare vascular compression syndrome in which the duodenum is compressed between the aorta and the overlying superior mesenteric artery. This condition is often chronic and secondary to cachexia. It can trigger further weight loss due to the subsequent proximal intestinal obstruction, causing a positive feedback loop. We report a case of acute presentation of superior mesenteric artery syndrome, complicated by gastric necrosis and treated surgically using the principles of a novel bariatric procedure.

## Introduction

Superior mesenteric artery (SMA) syndrome, also known as Wilkie’s syndrome, is a rare cause of proximal intestinal obstruction with an estimated prevalence of 0.1% to 0.3% [1]. It is caused by a decrease in the aortomesenteric (AM) angle and AM distance, leading to a compression of the third part of the duodenum (D3). The normal range of the AM angle and distance is 28-65 degrees and 10-34 mm, respectively [2]. Symptoms typically arise when the angle decreases to 22 degrees or less and the distance between the aorta and SMA is less than 8 mm. It is often preceded by significant weight loss, causing a loss of the mesenteric fat pad between the aorta and SMA, which, in turn, causes a narrowing of the AM angle. It should be noted that radiographic findings alone are not sufficient as criteria, as symptoms should be present to warrant the diagnosis of SMA syndrome [1,2].

The presentation can be either acute or gradually worsening with signs and symptoms of high intestinal obstruction such as epigastric pain which worsens in the supine position and alleviates in the left lateral decubitus position, abdominal distention, nausea, and vomiting [1,3]. In turn, complications such as dehydration, metabolic disturbances, and aspiration pneumonia may follow [3].

Diagnosis can be made by a variety of imaging modalities. However, the standard for diagnosis remains computed tomography (CT), in which the diagnosis can be confirmed by measuring the AM angle and distance on sagittal reconstructions. Furthermore, possible complications, such as gastric necrosis, or other causes of high intestinal obstruction, such as tumoral or inflammatory lesions can be detected as well [2].

Conservative treatment with fluid resuscitation, nasogastric tube placement, nutritional support, and postural therapy has its place in non-complicated cases as a means to restore the AM fat pad and thereby de-obstruct the duodenum. Failure of these conservative measures or acute presentation with severe symptoms or signs of ischemia may lead to a need for surgical management. This can be accomplished through either duodenojejunostomy, gastrojejunostomy, or repositioning the duodenum to the right side of the SMA after the division of Treitz’s ligament, known as Strong’s procedure [3,4].

## Case presentation

A 20-year-old male with a previous medical history of Prader-Willi syndrome, insulin resistance, hypogonadism, behavioral disorder, and bilateral mastectomy for gynecomastia presented to the emergency department (ED) of a general hospital in Antwerp, Belgium, with complaints of abdominal pain and signs of intestinal obstruction for one day. Recent history was relevant for intentional rapid weight loss of approximately 50 kg in less than six months through dietary modifications, resulting in a body weight of 48 kg. Both the patient and parents denied a recent history of binge eating. Metformin, testosterone, growth hormone, and risperidone were noted as chronic medications.

Physical examination at the ED showed a patient with signs of dehydration in hypovolemic shock with a heart rate of 148 beats per minute and hypotension of 48/20 mmHg. Abdominal examination showed no previous surgical scars on a distended abdomen with rebound tenderness.

A full blood workup was significant for a white blood cell count of 19 × 10^9^/L, creatinine of 1.49 mg/dL, and lipase of 940 U/L. Furthermore, other laboratory test results, including hemoglobin, C-reactive protein, and liver tests were within the normal range.

**Figure 1:**
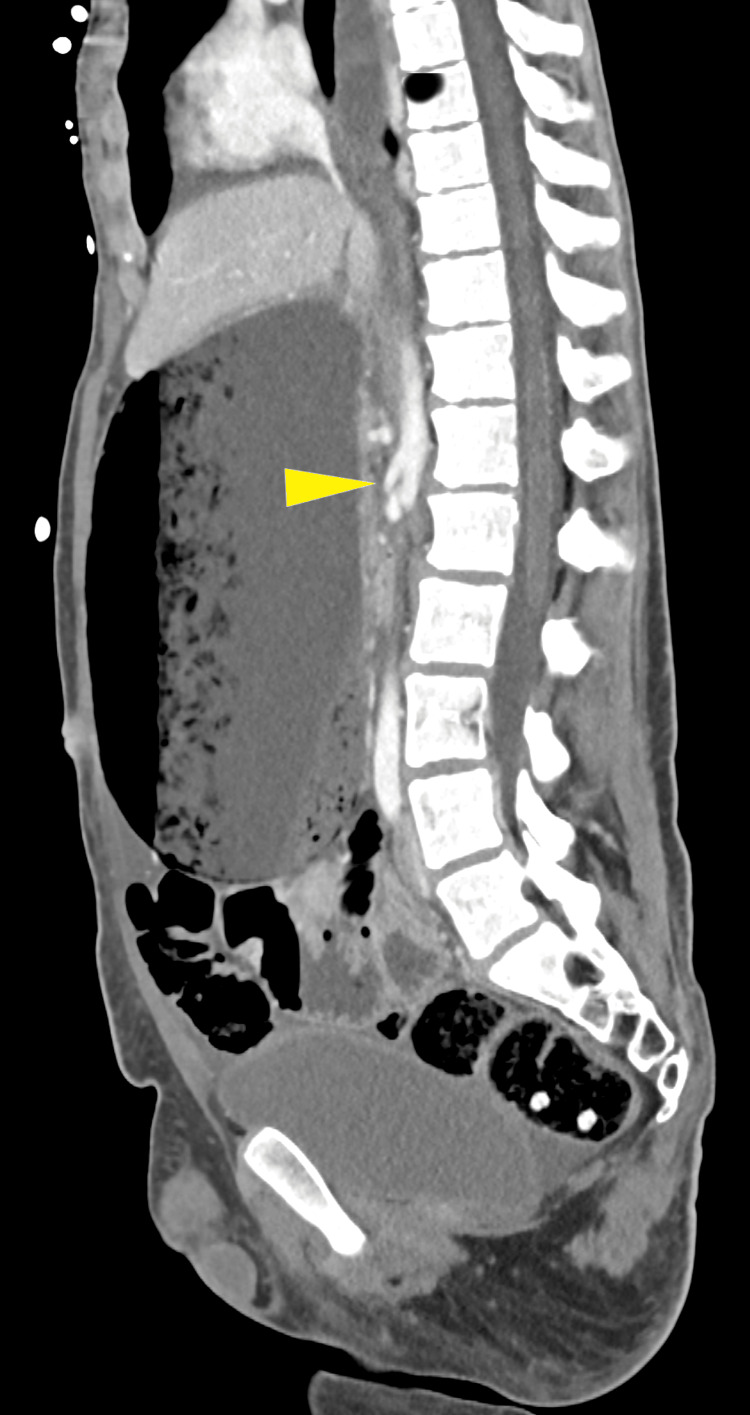
Sagittal view of the abdominal CT scan showing a narrowed AM angle. Yellow arrow: aortomesenteric angle. CT: computed tomography; AM: aortomesenteric

**Figure 2:**
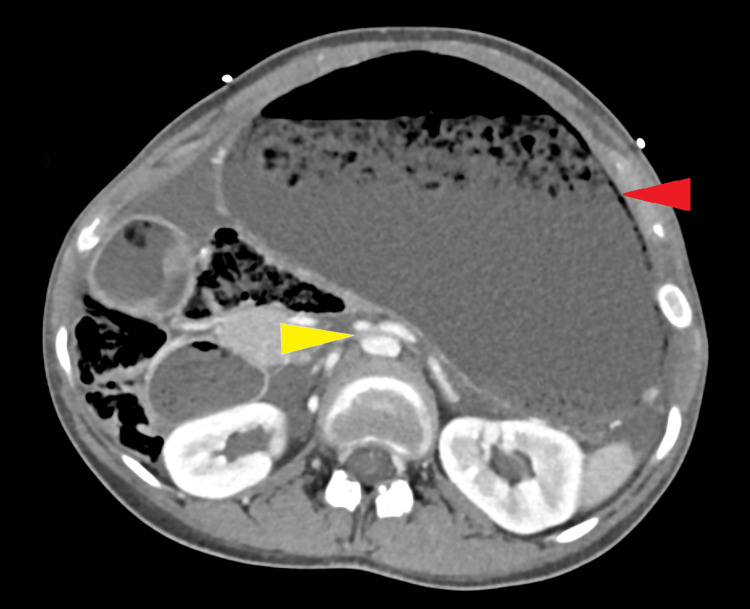
Axial view of the abdominal CT scan showing a reduced AM distance and gastric distention with pneumatosis Yellow arrow: aortomesenteric distance. Red arrow: gastric pneumatosis in the distended stomach. CT: computed tomography; AM: aortomesenteric

A CT scan of the abdomen with intravenous contrast was performed and revealed SMA syndrome, complicated with a pathologic distention of the esophagus, stomach, and duodenum up to D3. The AM angle measured 7 degrees and the AM distance was 4.3 mm, suggesting SMA syndrome. Secondary findings included pneumatosis of the stomach wall, pneumobilia, and ascites (Figures 1, 2).

Prompt fluid resuscitation was initiated along with pain management and antibiotic treatment. A nasogastric tube was inserted, draining 3.5 L of bilious fluid.

**Figure 3:**
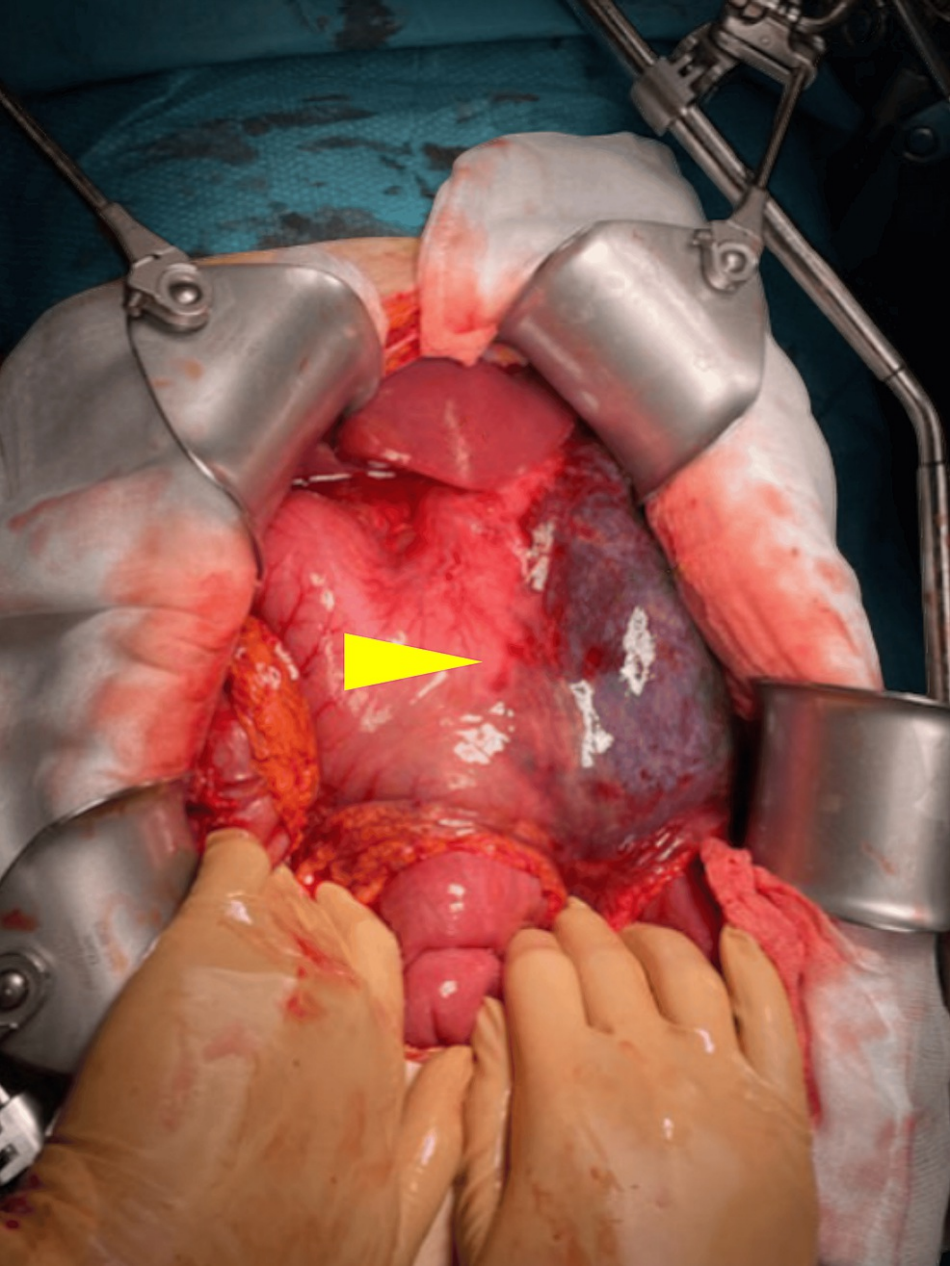
Peroperative view during urgent laparotomy. Yellow arrow: well-defined border of left lateral gastric wall necrosis on the distended stomach.

The patient was admitted and instantaneously transferred to the operating room where midline laparotomy was performed. The SMA could be felt spanning tautly over D3, confirming the diagnosis of SMA syndrome. The left lateral stomach wall along the greater curvature was necrotic with a well-defined border. The necrosis included the left lateral part of the fundus and corpus, without defects in the intraluminal space (Figure 3).

**Figure 4:**
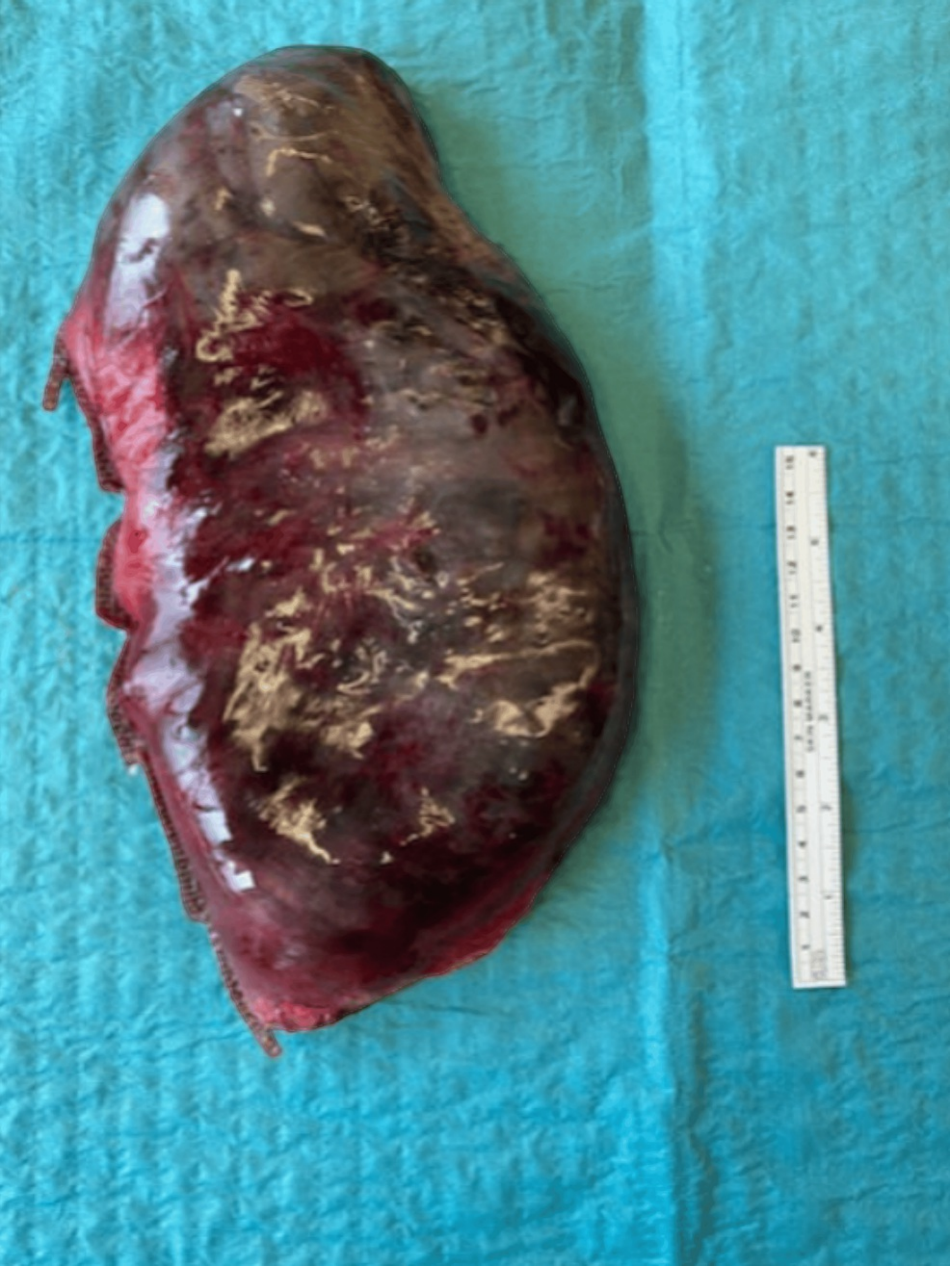
Specimen of the necrotic left lateral stomach after stapled sleeve gastrectomy. A 15 cm ruler for comparison.

A sleeve gastrectomy of the necrotic part (Figure 4) was performed using a GIA™ 6038S linear stapler (Covidien, Medtronic, Dublin, Ireland).

**Figure 5:**
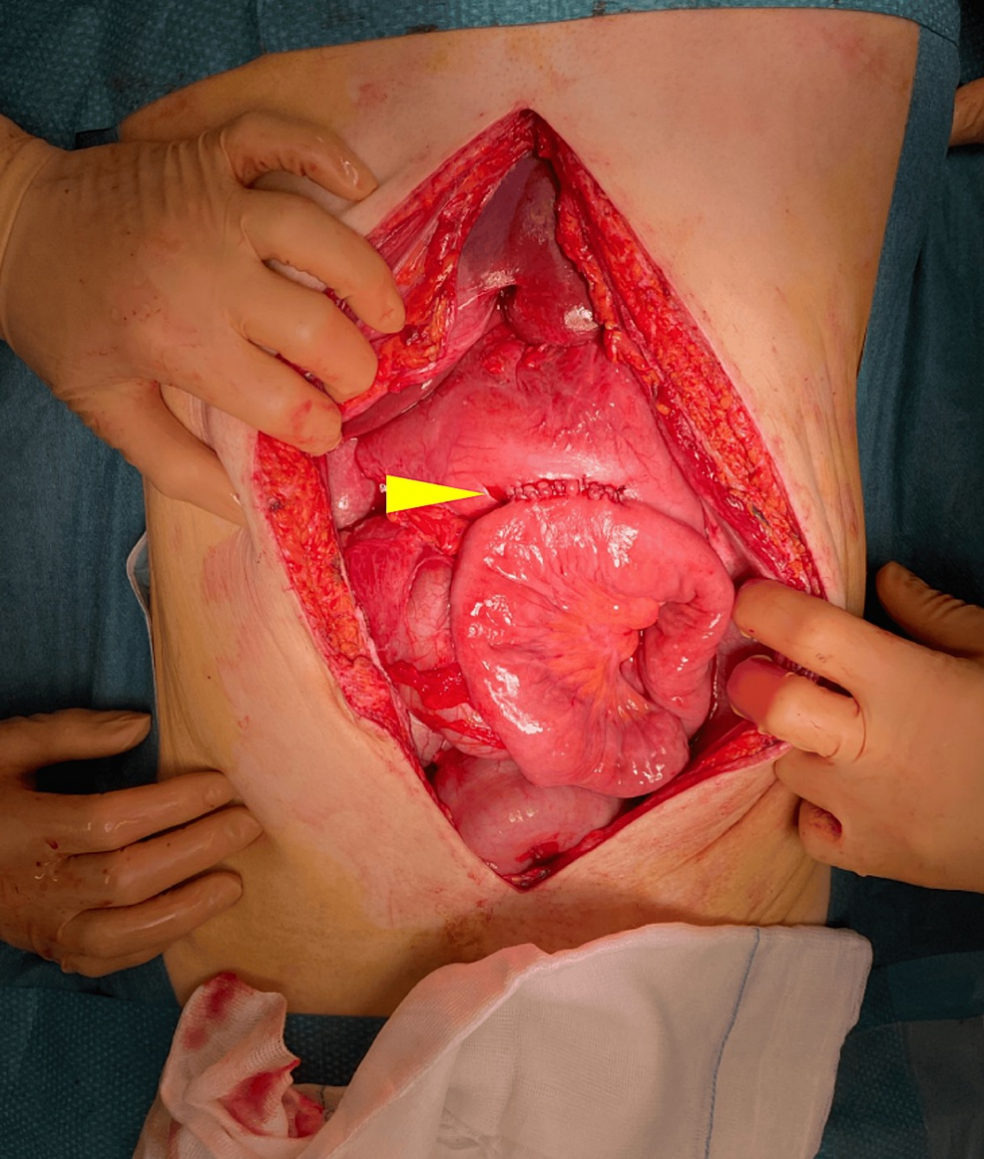
Peroperative view during the second operation after finishing the gastrojejunostomy of the SASJ reconstruction. Yellow arrow: gastrojejunostomy. SASJ: single anastomosis sleeve gastrectomy with jejunal bypass

Due to the hemodynamic and biochemical state of the patient, the operation was done in a two-step fashion, performing the anastomosis during a relook laparotomy after a 24-hour interval of further stabilization in the intensive care unit (ICU). During the second step, an intact staple line was observed, with no further expansion of the previously present signs of ischemia. It was, therefore, deemed appropriate to perform an anastomosis, which was performed as an omega loop gastro-jejunostomy starting 50 cm from Treitz’s ligament (Figure 5), resulting in a single anastomosis sleeve gastrectomy with (a limited) jejunal bypass (SASJ), a non-bariatric variation on the classic SASJ. Performing a duodenojejunostomy was initially considered, but the idea was ultimately abandoned due to sequelae of the grossly distended duodenum being present, indicating a heightened risk for anastomotic leakage, and given the surgeon’s limited expertise in duodenal surgery.

Postoperatively, the patient was started on total parenteral nutrition as well as proton pump inhibitors (PPIs). The patient was discharged from the ICU to the surgical ward on day one and the nasogastric tube was removed on day five after an upper gastrointestinal series confirmed patency of the anastomosis and absence of leakage. On day eight, the patient was discharged from the hospital in a generally well condition. Continued use of PPIs was advised for at least three months postoperatively.

A follow-up consultation at six months postoperatively showed an absence of abdominal complaints. The patient regained the possibility of enjoying all types of food, resulting in a weight gain of 7 kg. Control gastroscopy showed mild erosive gastritis, for which PPIs were restarted. The dose of metformin was reduced. A second gastroscopy one year later showed normal mucosa, without residual signs of gastritis and an uncomplicated anastomosis. The patient’s weight remained stable and PPI use could be discontinued.

## Discussion

A review of the literature shows no clear consensus on the ideal management of SMA syndrome in acutely complicated presentations. However, similar case reports have been published, describing different outcomes, ranging from successful surgical intervention with partial or total gastrectomy to death [3,5,6].

Upon presentation of a case of Wilkie’s syndrome, complicated by gastric necrosis, it is crucial to differentiate between mucosal and transmural necrosis. Multiple case reports have been published showing a possible conservative treatment in patients where necrosis is confined to the gastric mucosa, without transmural extension [7,8]. A CT scan can reveal signs of gastric ischemia or necrosis, demonstrated by pneumatosis of the gastric wall, portal venous air, or free air in case of perforation. In hemodynamically stable patients with suspected gastric necrosis, but without radiological or clinical signs of perforation, an endoscopic evaluation to assess the extent of ischemia seems helpful in determining the need for surgical intervention [9-11].

Prader-Willi syndrome (PWS) is a rare genetic disorder caused by a lack of expression of genes on the paternally inherited chromosome 15, which leads to multiple endocrine, metabolic, and behavioral disorders. The estimated prevalence ranges from 1 in 10,000 to 1 in 30,000 individuals. Patients with PWS typically present with hypotonia, poor feeding, and failure to thrive in infancy. In childhood, however, the eating pattern tends to turn into hyperphagia with uncontrolled appetite, leading to a prevalence of overweight and obesity of 40% in children and adolescents and 80% to 90% in adults [12,13].

Acute gastric dilation in PWS has been documented. A case series by Wharton et al. described acute gastric dilation complicated by gastric necrosis in six patients with PWS. One patient, a 36-year-old woman, showed significantly similar characteristics to our case. She presented with abdominal pain, vomiting, and signs of circulatory shock, with a recent weight loss of 45.5 kg during the five preceding years. A CT scan showed a distended stomach and duodenum up to D3. Exploratory laparotomy was performed in which a stomach with extensive necrosis was found and a subtotal gastrectomy with gastrojejunostomy was done. Although not explicitly mentioned in the article, it seems plausible this patient suffered the same fate as ours, with SMA syndrome as the underlying pathology [14].

A high pain threshold and the inability to vomit have been considered as features predisposing patients with PWS to gastric dilation [14]. Arenz et al. measured gastric emptying in eight patients with PWS using nucleotide scintigraphy and showed prolonged half-time values in five. Their findings suggest that delayed gastric emptying could be an additional contributing factor for acute gastric dilation in patients with PWS [15].

It seems plausible that the combination of PWS and the recent rapid weight loss have both contributed to the occurrence of SMA syndrome complicated with gastric necrosis in this patient.

In this case, a gastrojejunostomy was preferred over a duodenojejunostomy. Duodenojejunostomy has been proven to be a safe and viable option and is proclaimed to be the preferred method of treatment for SMA syndrome, with a success rate of 90%. Though gastrojejunostomy is often listed as a possible technique, it does have its disadvantages. It has been reported to fail to completely resolve duodenal dilatation and is associated with complications such as dumping syndrome, bile reflux, anastomotic ulcer, and blind loop syndrome [4,16]. Failure of gastrojejunostomy could eventually lead to the need for conversion to a duodenojejunostomy. Although no randomized controlled trials comparing duodenojejunostomy with gastrojejunostomy in SMA syndrome have been published to date, it is generally recommended to reserve gastrojejunostomy for situations in which the duodenum is deemed unsuitable for anastomosis only [17].

SASJ is a relatively new surgical technique used in bariatric surgery. Multiple studies have shown it to be a safe and effective strategy in weight loss management, with an acceptable risk for major complications [18]. However, due to the novelty of this procedure, further long-term studies are needed to evaluate safety after longer periods of follow-up, especially as there is a concern for bile reflux with these types of intestinal reconstructions [19].

## Conclusions

Gastric necrosis following acute gastric dilatation is a rare complication of SMA syndrome with varying outcomes and no clear consensus on the ideal treatment. A high level of vigilance should be maintained, especially in patients with a history of eating disorders, rapid weight loss, or PWS. The diagnosis can be made based on CT findings. Management options range from conservative to surgical and should be based on both patient characteristics and local expertise. In the case at hand, an open two-stage procedure with sleeve gastrectomy, followed by gastrojejunostomy, seems to have been a life-saving intervention, with a favorable outcome after one and a half years of follow-up.
